# Looking forward to a decade of the biopsychosocial model

**DOI:** 10.1192/bjb.2022.34

**Published:** 2022-08

**Authors:** Derek Bolton

**Affiliations:** 1 King's College London, UK

**Keywords:** Biopsychosocial model, biomedical model, George Engel, biopsychosocial health sciences, biomedically unexplained symptoms

## Abstract

The topic of this article is the biopsychosocial model. My main contention is that – notwithstanding doubts as to what exactly it is, or indeed whether it is anything – there is a coherent account of it, in terms of both applications to particular health conditions and mechanisms with wide application. There is accumulating evidence from recent decades that psychosocial as well as biological factors are implicated in the aetiology and treatment of a large range of physical as well as mental health conditions. The original proposer of the biopsychosocial model, George Engel, back in 1977, was substantially correct about what he saw was on its way.

## Recent controversies around the biopsychosocial model

In conversations where the biopsychosocial model comes up, comments are commonly heard to the effect of: ‘Well, we use it and teach it, but we don't know what it is!’ The problem that we don't know exactly what the model is naturally gives rise to the worry that it isn't anything, and a decade or so ago this worry was being voiced loudly and clearly by experts in medicine generally and psychiatry in particular.^1–4^

Although at first sight it is puzzling that we should use and teach something without knowing what it is, we can bear in mind that the biopsychosocial model has to do with many or all types of health conditions, professions and specialties, and so we should hardly expect it to be *simple*. In fact it's more likely to be complicated. Even if it were assumed that George Engel knew what the model was when he first proposed it in his 1977 paper,^5^ that was nearly half a century ago, and clinical sciences and services have all changed a lot since then. So what, if anything, do we mean by it now?

## Recent work on the biopsychosocial model: theory and applications to specific conditions and stages

Since the vocal criticisms of the biopsychosocial model a decade or so ago, referred to above, there has been new work on the model, including a monograph^6^ (with subsequent commentaries^7–12^) and an edited volume.^13^

Further, there have continued to be many references to the biopsychosocial model, or biopsychosocial factors, in published studies of specific health conditions. Casual (non-systematic) web searches of the form ‘*biopsychosocial model of / factors for [name of a health condition]*’ generate lists of studies.^14–16^ Studies that invoke biopsychosocial factors, with or without explicit use of the term ‘biopsychosocial model’, typically refer to psychosocial as well as biological factors in aetiology, course, treatment, adjustment and/or quality of life.

Particularly relevant in the present time of the COVID-19 pandemic are the clear demonstrations of the roles of multiple factors – biological, psychosocial and sociopolitical – that combine in complex ways to determine exposure, vaccination status, population prevalence, individual caseness, course, mortality, and longer-term recovery and quality of life.^17–19^ This recent work suggests that the biopsychosocial model of at least specific health conditions is alive and well; moreover, it is even used for modelling infectious diseases, not only non-communicable conditions.

Further, the fact that the biopsychosocial model is thriving in specific applications suggests a response to the worry, highlighted above, that even though we use and teach the model, we don't know what it is, and perhaps it isn't anything. The response is that when using or teaching the model we do so with particular conditions and stages in mind, along with supporting data. To note, much the same would apply to using and teaching any other general model of health and disease, including the one the biopsychosocial model is usually contrasted with, the ‘biomedical model’.

## What is the ‘general model’?

Insofar as the content and utility of the biopsychosocial model lies in its application to specific conditions, at specific stages, what is the point of the (or a) general model? As just noted, the biopsychosocial model is usually contrasted with the ‘biomedical model’. But then, also, what exactly is the biomedical model? Probably all such short expressions about models or any terminology alluding to a general approach, such as ‘biological psychiatry’ or ‘neuroscience-based psychiatry’ are ambiguous, without clear content – how could they be otherwise, being so short? Therefore, they run the risk of being no more than memorable phrases, something like ‘slogans’.

An alternative way of looking at this kind of terminology is that the terms serve as shorthand for methodological assumptions or hypotheses as to where causes and cures will be found, along with whatever evidence supports them.

In this spirit, I suggest that we can make use of the term ‘biopsychosocial model’ as a shorthand for methodological assumptions that causes and/or cures of specific conditions at specific stages, including matters of adjustment and quality of life, will generally – across a wide range of conditions – include biological, psychological and social factors, and interactions between them. The contrast is then with the ‘biomedical model’, which deals with biological factors only.

Going further, it is possible to construct more theorised versions of general models or orientations that underpin practice. These theorised general models include foundational-level characterisation of the relevant domain(s) and causation within them, and between them if more than one, in function and dysfunction. Thus, a theorised version of the biomedical model includes core concepts and principles/models of the biomedical sciences. Similarly, a theorised biopsychosocial model would include core concepts and principles/models of the biological, psychological and social sciences relevant to health and disease.^6^ Two examples of foundational biopsychosocial theories are outlined in the next section, and some key theoretical aspects of the relations among psychology, psychiatry and neuroscience are briefly described later in the paper.

## Two examples of core theory in biopsychosocial health science

Two major new explanatory theories that integrate biopsychosocial factors across very wide ranges of health conditions have been developed in the past few decades: one implicates *chronic stress* and the other *central involvement in pain perception*. Both can be accommodated within the general biopsychosocial model – and, to be clear, not within the general biomedical model – illustrating how the general biopsychosocial model has applications not only to specific conditions but also transdiagnostically across a very wide range. The new theories are well-known in the literature, and I will summarise them here only to highlight their relevance to the biopsychosocial model.

The chronic stress model has been developed in explanatory epidemiology to link social determinants of health to a range of adverse health outcomes. Core interlinked pathways include the following: chronic psychological stress results from chronic lack of control over salient outcomes; chronic lack of control is associated with low resource levels (such as working poverty); it raises risk of anxiety and depression; chronic physiological arousal associated with chronic psychological stress raises the risk of immunological dysregulation and biological damage.^20,21^ The chronic stress model (or set of related models) and associated data are thoroughly biopsychosocial and represent major discoveries about the aetiology of many health conditions, especially non-communicable diseases – in some ways comparable to the great biomedical model of infectious diseases.

The second example of a new explanatory biopsychosocial theory with very wide application is about pain perception, that it involves neurobiological and psychological factors as well as peripheral physiological or structural damage. The new models of pain perception implicate the person's negative appraisals of what their pain means and the expected adverse effects on their lives, and associated central nervous system pain-processing mechanisms.^22,23^ This new understanding of pain is at least neurobiopsychological, and it includes psychosocial factors to the extent that (perceived) adverse effects of pain on people's lives depend on the social context and task demands.

This new understanding of pain perception is directly relevant to conditions dominated by pain, but there is a much broader point that is relevant to the health sector as a whole, specifically to drivers of service use. The new models of pain perception incorporate pain, plus distress about pain, plus associated impairment of functioning; this complex of negative mental state and downturn in behavioural functioning is a close approximation to people ‘feeling unwell’ and is a main driver of referral and service use. This has implications for general medicine to be considered below.

## Implications for general medicine

The complex of pain, distress and associated impairment is a common presentation in general medicine; importantly, however, in a significant proportion of such presentations, biomedical investigations show no or insufficient detectable biological damage. In such cases, in some contexts, the presenting symptoms are called ‘medically unexplained symptoms’ (MUS), which means here ‘*biomedically* unexplained’. However, and this is the main issue, biopsychosocial management for these cases is indicated by the new biopsychosocial models of pain perception but is not routinely provided.

The large scale of the problem is well-known. In a recent review, Jadhakhan and colleagues^24^ summarise as follows (citations omitted):

*It is estimated that MUS accounts for approximately 20% of new consultations in primary care, 52% of new referrals in secondary care and 20%–25% of all frequent attenders at medical clinics. Patients with MUS are commonly referred for multiple investigations and assessments with little benefit, so are needlessly costly for healthcare systems and account for approximately 10% of the total National Health Service (NHS) expenditure for the working-age adult population in England.*

Specialties in which presentations of pain, distress and impairment with no or insufficient biomedical explanation arise include cardiology,^25–27^ neurology,^28^ and surgery for some pain presentations compared with placebo.^29–31^ The implication is that a broader medical approach is required, and the new models of pain, distress and associated impairment suggest that this should include attention to biopsychosocial factors.

## A decade of the biopsychosocial model?

The question arises whether the new paradigms and findings of the sort reviewed above warrant a decade of the biopsychosocial model. The reference here is of course to ‘the decade of the brain’ in the 1990s. This followed from the development of new neuroscience technology in the 1980s, was instigated by the US Congress, and included increased funding for and public education on neurological and some psychiatric conditions and new technology.^32,33^

Correspondingly, a decade of the biopsychosocial model would include increased publicity on and funding for research on biopsychosocial aetiology and prevention programmes, to include further research on social determinants of health and on mechanisms linking indices of social exclusion, chronic stress and illness, and on the identification of modifiable prevention targets. Such initiatives would come under the heading of *biopsychosocial aetiology and prevention*. Under the heading of *illness, diagnosis and treatment*, there could be increased publicity on and funding for research on pain, distress and impairment as drivers of service use across many medical specialties and on appropriate biopsychosocial management and treatment provision.

Psychiatry already includes biopsychosocial approaches, especially but not only in the context of multidisciplinary teams. Psychosocial formulations and treatments are already typical in mental health services. So what would a decade of the biopsychosocial model look like for psychiatry? An obvious point is that the ‘biological’ in psychiatry involves neuroscience, in contrast with the biomedical sciences that underpin biomedicine; in this sense, psychiatry has already been catered for in ‘the decade of the brain’. Arguably, however, neuroscience so far – including in that decade – has not advanced psychiatry much; see, for example, David Kingdon's 2020 paper^34^ with the polemical title: *Why hasn't neuroscience delivered for psychiatry*? Kingdon calls for more research into psychosocial factors in psychiatry, and this is surely the right call.

On these issues, however – on connections between psychiatry, neuroscience and the biopsychosocial – I would emphasise that neuroscience is properly understood in a broad way to include psychological and behavioural functioning (see, for example, the neuroscience programmes at Harvard (https://www.mcb.harvard.edu/undergraduate/neuroscience/) and UCL (https://www.ucl.ac.uk/research/domains/neuroscience). This broad conceptualisation of neuroscience includes models of ‘embodied cognition’, that is, cognition that is environmentally involved.^35,36^ The implication is that psychiatry should be concerned not only with neurobiological pathology but also with the vagaries of psychological processing and with environmental interactions.^37,38^ It can be noted here that the importance of including environmental interactions in a broad understanding of neuroscience was recognised in the ‘decade of the brain’, which included increased funding for early childhood prevention programmes given emerging evidence of adverse early environmental effects on brain development.^32,33^

## Psychiatry and ‘the rest of medicine’

Another context for the questions of what a decade of the biopsychosocial model would be like for medicine generally and for psychiatry particularly is that in Engel's original formulation, the relation between psychiatry and ‘the rest of medicine’ was a kind of proxy for the relation between the biomedical and the biopsychosocial models. This is how Engel starts his 1977 paper:^5^

*At a recent conference on psychiatric education, many psychiatrists seemed to be saying to medicine, ‘Please take us back and we will never again deviate from the “medical model”’. For, as one critical psychiatrist put it, ‘Psychiatry has become a hodgepodge of unscientific opinions, assorted philosophies and “schools of thought,” mixed metaphors, role diffusion, propaganda, and politicking for “mental health” and other esoteric goals’. In contrast, the rest of medicine appears neat and tidy. It has a firm base in the biological sciences […] and a record of astonishing achievement in elucidating mechanisms of disease and devising new treatments*.

Engel is on his way to recommending the biopsychosocial model, with the implication that psychiatry's involvement with the psychosocial and not only the biological puts it ahead of the curve, not behind.

At the start of his paper he sets up the ‘medical model’ as a target, but more precisely his target is medicine ‘with a firm base in the biological sciences’ (as in the above quote), i.e. biological medicine (biomedicine) and the ‘biomedical model’. This is consistent with the full title of the paper – ‘The need for a new medical model: a challenge for biomedicine’ – and Engel soon switches to this terminology of *biomedical* and *biomedicine*. To some extent this is a terminological issue, but more importantly it leaves open the possibility that medicine and its ‘medical model’ have always been biopsychosocial and to a great extent still are. On this point, biomedicine is not itself a medical specialty but rather a class of biological models of health and disease and associated technology for prevention, detection and treatment, which are more or less applicable in particular medical specialties. General practice and some medical specialties such as psychiatry are well-known to need a broader biopsychosocial approach. If anyone had any doubt that the management of public health requires biological–psychological–behavioural–social(–political–economic) modelling, this would have been removed by what we have seen in the current COVID-19 pandemic. However, as indicated above, accumulating evidence suggests that even medical specialties with records of astonishing biomedical achievements need a biopsychosocial extension to accommodate significant proportions of their patients.

Continuing to develop the main point, Engel at the start of his paper turns the criticism of psychiatry – that it uses biopsychosocial management as opposed to the precision of the rest of medicine using biomedical management – on its head. To continue the quote from Engel's paper^5^ (p.129; text in brackets added):

*It would seem that psychiatry would do well to emulate its sister medical disciplines by finally embracing once and for all the [bio]medical model of disease.*

*But I do not accept such a premise. Rather, I contend that all medicine is in crisis and, further, that medicine's crisis derives from the same basic fault as psychiatry's, namely, adherence to a model of disease [i.e. the biomedical model] no longer adequate for the scientific tasks and social responsibilities of either medicine or psychiatry.*

In this paper I have tracked Engel's approach. Rather than special pleading for psychiatry, or calls for it to be exclusively biological, like (bio)medicine, or an applied physico-chemical neuroscience that doesn't include psychological and psychosocial factors, I have highlighted medicine's apparent need to be more biopsychosocial, like psychiatry.

## About the author

**Derek Bolton** is Emeritus Professor of Philosophy and Psychopathology at the Department of Psychology, Institute of Psychiatry, Psychology and Neuroscience, King's College London, UK.
